# Towards Symmetric Thioamides: Microwave-Aided Synthesis of Terephthalic Acid Derivatives

**DOI:** 10.3390/ph16070984

**Published:** 2023-07-09

**Authors:** Andrzej Bak, Violetta Kozik, Aleksandra Swietlicka, Wojciech Baran, Adam Smolinski, Andrzej Zięba

**Affiliations:** 1 Institute of Chemistry, University of Silesia, Szkolna 9, 40-006 Katowice, Poland; aswietlicka@us.edu.pl; 2 Department of General and Analytical Chemistry, Faculty of Pharmaceutical Sciences in Sosnowiec, Medical University of Silesia in Katowice, Jagiellońska 4, 41-200 Sosnowiec, Poland; wbaran@sum.edu.pl; 3 Central Mining Institute, Plac Gwarków 1, 40-166 Katowice, Poland; smolin@gig.katowice.pl; 4 Department of Organic Chemistry, Faculty of Pharmaceutical Sciences in Sosnowiec, Medical University of Silesia in Katowice, Jagiellońska 4, 41-200 Sosnowiec, Poland; zieba@sum.edu.pl

**Keywords:** thioamides, terephthalic acid, thionation, microwave-accelerated synthesis, *bis*-terephthalthioamides

## Abstract

The multistep synthesis of novel *bis*-terephthalthioamides based on methyl esters of amino acids (AAs) was proposed using conventional heating and microwave-assisted approaches. In fact, the comparative case study on the thionation of new symmetrical diamides with Lawesson’s reagent (LR) was performed. The microwave-accelerated small-scale methodology was successfully employed on the whole pathway from substrates (Gly, Ala, Val, Tyr, Ser) to products (symmetrical dithioamides of terephthalic acid), resulting in significantly reduced reaction time, energy requirements, and slightly increased reaction yields when compared to conventional heating. Moreover, the intermolecular similarity of novel terephthalic acid derivatives was estimated in the multidimensional space (mDS) of the structure/property-related in silico descriptors using principal component analysis (PCA) and hierarchical clustering analysis (HCA). The distance-oriented structure/property distribution was also correlated with the experimental lipophilic data.

## 1. Introduction

Amino acids (AAs) play a crucial role in many vital processes of living organisms; therefore, (bio-)transformation of AAs seems attractive from the point of the ‘rational production of properties’ in medicinal chemistry [1,2,3,4]. The incorporation of AAs, either natural or their derivatives (e.g., esters or amides), as components of the parent (pro-)drugs is basically regarded as a patient-friendly approach on the route from structures to ADMET-tailored properties [5,6]. Due to their low toxicity, AAs can also be attractive drug carriers for poorly absorbed curative agents on the pathway from the place of administration to the site of interaction. In fact, the carboxylic/amine substituents attached to α-carbon are usually the linkers of the parent drug that abolish the zwitterionic form [7]. In practice, AA-drug linkage chemistry is well established in producing clinically used therapeutic agents (e.g., L-Val-gemcitabine) [8,9,10]. 

**Figure 1:**
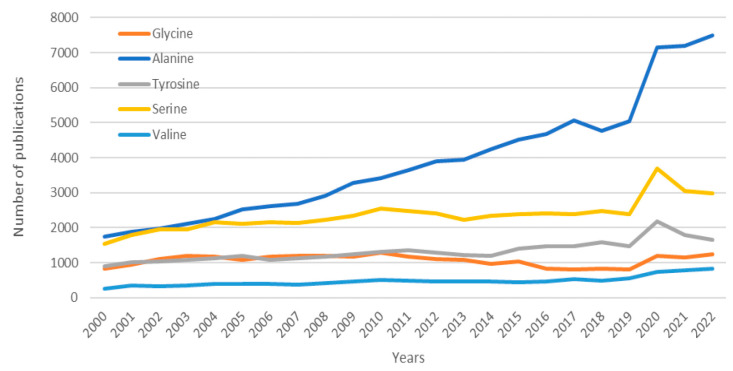
The number of papers published in medicinal chemistry, where the name of the selected AAs was searched in the paper title or abstract according to Reaxys data.

Due to the unique chemical character of α-AAs, they hold the permanent interest of the scientific community, which can be illustrated by the number of reports published in the field of medicinal/pharmaceutical chemistry, as shown in Figure 1. Hence, the extensive database screening of the Reaxys repository was performed to identify the hits, where the name of the selected α-AAs (R = Gly, Ala, Tyr, Ser, Val) was queried in the title or abstract of the papers published in the last two decades (from 2000 to 2022). Generally, the constant growth in the number of publications related to α-AAs has been recorded in the period of the last 20 years, with visible ‘peaks’ of interest reached during the SARS-CoV-2 pandemic. Naturally, the indicated ‘wavy’ trends in the α-AA-related scientific activity can partially correlate with the extensive efforts of academia and industry to deliver new antiviral drugs. 

Terephthalic acid (TPA) is an essential dicarboxylic intermediate massively manufactured commercially; therefore, the environmental fate and/or potential biological routes of TPA in living organisms, including adsorption, (bio)transformation, and excretion, need to be examined in detail [11,12]. Moreover, in vitro metabolic studies have revealed that TPA is basically a non-genotoxic/mutagenic chemical at normal doses with pretty low toxicity (LD_50_ > 5000 mg/kg by oral administration) [13]. Interestingly, the successful conversion of poorly water-soluble antifungal itraconazole (ITR) into the multi-component solid phase (cocrystals) was conducted using TPA as an aromatic coformer [14,15]. 

The poly(ester amide) containing α-AA-based motifs is of great interest as an attractive material in the biomedical field and tissue engineering [16]. In fact, the formation of the amide bond is among the most prevalent transformations incorporated in the large-scale production of active pharmaceutical ingredients (APIs) in medicinal chemistry [17,18]. In the bioactive molecules (e.g., proteins, DNA, or RNA), nature itself has encoded the unique features of the amide bond, its high stability towards various reaction conditions (e.g., pH, temperature), and its ability to form resonating structures and characteristic spatial atomic distribution, which hinder the free atom rotations within the HN-C=O motif [19,20]. 

**Figure 2:**
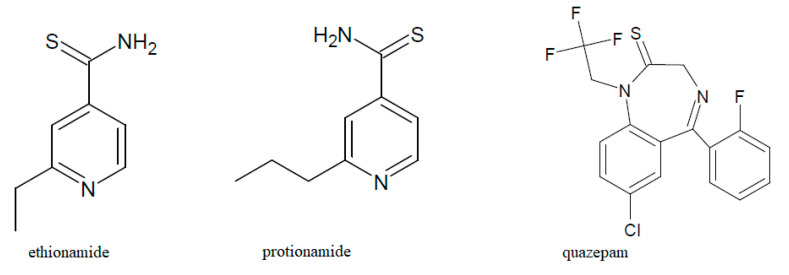
Thioamide-based approved therapeutic agents according to DrugBank database.

Overall, organosulfur compounds are valued in pharmacology as antitumor, antimicrobial, anti-HIV, and chemoprotective agents against a variety of carcinogenic or toxic factors [21,22,23]. The single-atom substitution of the carbonyl oxygen in an amide bond with sulfur (HN-C=S) is generally regarded as an isosteric replacement to produce more potent and stable molecules with modified bioactive potency (e.g., the antibacterial activity) [24,25]. Although amides and thioamides are somewhat similar structurally to each other, these two species vary noticeably in their chemical and physical properties—thioamides are more polar with a more decisive dipole moment [26]. The conversion of the amide-containing compounds into thioamides can also improve the ADMET properties [27,28,29]. The site-specific oxoamide→thioamide modification of proteolytically sensitive amide bonds in the peptide backbone is a widespread synthetic practice commonly used to specify the spatial distribution, stability, and functional properties of peptides, respectively. As a matter of fact, the validity of thioamide-based compounds was confirmed in medicinal applications (see Figure 2), revealing the bioactive potential of the thioamide motif (e.g., second-line antituberculosis and leprosy drugs) [30,31,32,33,34]. 

The principal objective of the presented study was the conceptual design and practical synthesis of the symmetrical α-AA-based (R = Gly, Ala, Val, Tyr, Ser) dithioamides of terephthalic acid using the conventional heating (method I) as well as the microwave-accelerated approach (method II). It was revealed that both methods yielded comparatively at each stage of the proposed three-step procedure. A noticeably reduced reaction time (from days to minutes) in the microwave-supported approach makes the method an attractive and eco-friendly alternative to the lengthy methodology of conventional heating. Following the common practice, the intermolecular similarity of novel terephthalic acid derivatives was estimated in the multidimensional space (mDS) of the structure/property-related in silico descriptors using principal component analysis (PCA) and hierarchical clustering analysis (HCA), respectively. Moreover, the distance-oriented property distribution for the new series of compounds was correlated with the experimental TLC and HPLC lipophilic data. Finally, the molecular structure of methyl 2-({4-[(1-methoxy-3-methyl-1-oxobutan-2-yl)carbamothioyl]phenyl}methanethioamido)-3-methylbutanoate (**5d**) was determined via single-crystal X-ray diffraction.

### Mini-Review of Thionating Reagents

A vast range of thionating agents, including elemental sulfur (S_8_), phosphorus decasulfide (P_4_S_10_), and Lawesson’s reagents (C_14_H_14_O_2_P_2_S_4_), have been used (either alone or in combination with additives) in the synthesis of organosulfur compounds. 

**Scheme 1:**
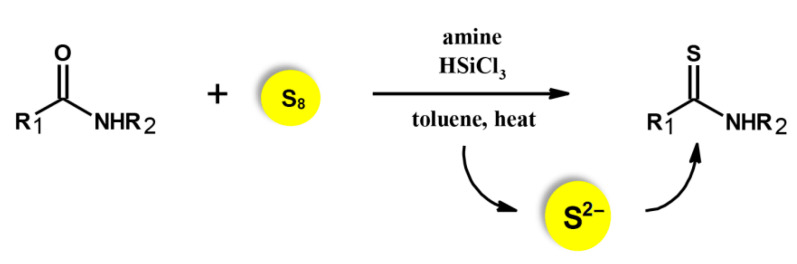
Direct thionation of amides by elemental sulfur using hydrochlorosilanes and amines.

In practice, the direct sulfurization of the carbonyl group using a preformed S^2−^ agent is notably cumbersome due to odor, toxicity, and stability issues [35]. Moreover, pretreatment of the carbonyl motif is frequently needed with the concurrent production of inseparable intermediates [36]. It was revealed that the specified reaction conditions, the choice of reducing agent, and the nature of the solvent are crucial factors of the thionation procedure. As shown in Scheme 1, easy-to-handle elemental sulfur (S_8_) was reduced in situ to S^2−^ in the presence of hydrochlorosilanes (as a reducing agent) and a suitable amine in order to generate the corresponding thioamides with acceptable yields [37]. 

**Scheme 2:**
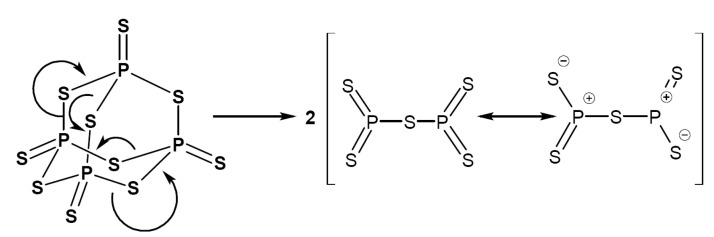
Spatial structure and dissociation mechanism of phosphorus decasulfide (P_4_S_10_).

The effective deoxygenation of carbonyls into the corresponding thiocarbonyls by means of crystalline solid phosphorus decasulfide (P_4_S_10_) was first introduced at the end of the XIX century [38]. P_4_S_10_ was successfully employed in 1878 to convert amides to thioamides by Hofmann [39,40]. It is suggested that the dimeric form of phosphorus decasulfide dissociates into corresponding monomers (phosphorus pentasulfide, P_2_S_5_), especially in the reactions conducted under refluxing solvents, as presented in Scheme 2. 

**Scheme 3:**
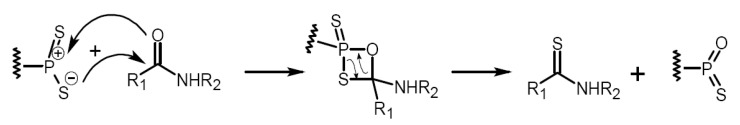
Mechanism of amide thionation via P_4_S_10_ agent.

A large excess of thionating reagent and lengthy reaction time were required in the environment of the boiling solvent (e.g., benzene, toluene, xylene, THF, CS_2_, CH_3_CN, or pyridine) to form thio-compounds [41,42]. Unfortunately, greenish-gray to yellow flammable crystals of P_4_S_10_ decompose rapidly on contact with water or even with atmospheric moisture, producing the odorous hydrogen sulfide (H_2_S) gas and phosphoric acid (H_3_PO_4_), respectively. Moreover, thionation of amides using P_4_S_10_ alone generates highly condensed polythiophosphates (see Scheme 3) as potential electrophiles (similarly to P_4_O_10_) that might promote unwanted side reactions of the carbonyl and thiocarbonyl compounds [43]. 

In recent years, new approaches that combine the conventional thionating reagents with additives (catalysts on a solid support) or novel techniques (microwave irradiation) have appeared in order to increase the product yields, simplify the purification processes, and/or optimize the reaction time/cost-consumption, respectively. A substantial improvement in P_4_S_10_ performance in thionation was reported, where the addition of hexamethyldisiloxane (Me_3_SiOSiMe_3_, HMDO) noticeably enhanced the utility of P_4_S_10_ in sulfurization of the carbonyl-based derivatives [44,45]. In particular, dichloromethane (CH_2_Cl_2_) was recommended as a useful solvent for the thionation of amides with the P_4_S_10_/HMDO combination; however, benzene can be applied at reflux temperature as well. Regrettably, the detailed mechanism of the beneficial impact that HMDO exerted on most of the P_4_S_10_-mediated thionations is not clear—these two components did not react directly with each other; therefore, a new, more effective thionating agent was not formed [46]. It is suggested that P_4_S_10_ first reacted with the carbonyl substrate and P_4_S_10_-derived intermediate, which subsequently reacted with HMDO to produce non-polar, soluble by-products (supposedly trimethylsilylated phosphates or thiophosphates) [47]. The presence of HMDO in the reaction environment probably converts potent electrophiles (condensed polythiophosphates) to innocuous silylated phosphates [39]. Actually, a simple hydrolytic workup or passage through silica gel can be employed to separate the undesired phosphorus-containing by-products during the purification process; however, the use of HMDO makes the method pretty cost-consuming [48].

The usability of the alumina-encapsulated P_4_S_10_ reagent (P_4_S_10_/Al_2_O_3_) as a solid-supported thionating agent in acetonitrile solvent (CH_3_CN) was investigated in the synthesis of long-chain thioamides and thioketones [44,48,49]. As the smooth reaction of amides/ketones with P_4_S_10_ proceeded, Al_2_O_3_ scavenged yield-lowering by-products formed in the course of thiocarbonylation, increasing the yield considerably [48]. It was noticed that the majority of by-products were anchored to the alumina; therefore, a simple filtration could be applied at the purification stage, omitting a cumbersome aqueous workup on the path to pure products. Furthermore, P_4_S_10_/Al_2_O_3_-mediated thionation of amides was effectively supported by microwave irradiation, which significantly shortened the reaction time (from approximately 6÷10 h to 5 min at a power of 60W) with a comparable yield achieved under conventional heating [49].

**Scheme 4:**
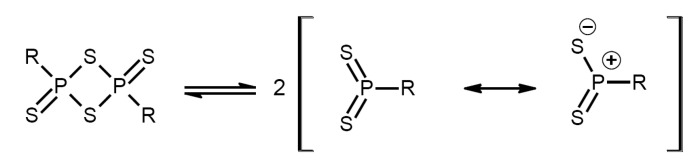
Lawesson (R = C_6_H_4_-OCH_3_), Belleau (R = C_6_H_4_-OC_6_H_5_), and Davy (R = SCH_3_) reagents.

In recent years, the 1,3,2,4-dithiadiphosphetane-2,4-disulfide family with the characteristic -P_2_S_2_- ring (see Scheme 4) has displaced P_4_S_10_ as the alternative thionating agent of choice in organic chemistry [50]. Technically, 2,4-*bis*(p-methoxyphenyl)-1,3,2,4-dithiadi-phosphetane-2,4-disulfide, a slightly yellow amorphous solid, was first synthesized by Lecher et al. in 1956 [51], but its versatile thionating potential was unraveled and popularized globally by a Swedish chemist, Sven-Olov Lawesson in 1978; therefore it is widely known as Lawesson’s reagent (LR) [52]. In solution, decomposition of the unique –P_2_S_2_– ring to 2 symmetric monomers was proposed; therefore, LR could coexist in equilibrium with more reactive dithiophosphine ylide, as illustrated in Scheme 4. 

**Scheme 5:**
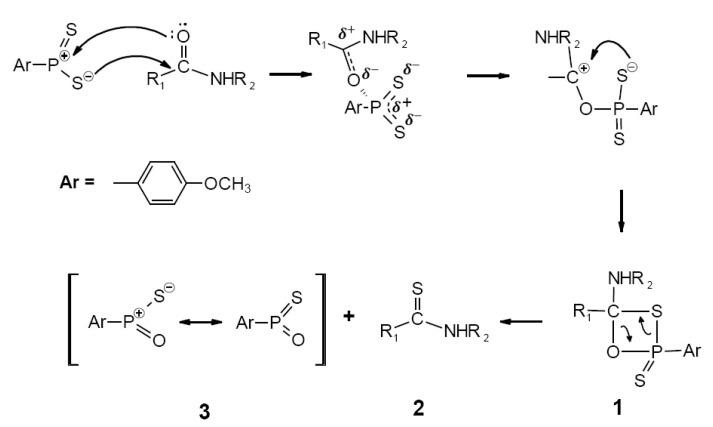
Mechanism of amide thiocarbonylation via Lawesson’s reagent.

As a matter of fact, the reactive monomeric form of the LR agent (4-methoxyphenylphosphine disulfide) has never been isolated or observed spectroscopically due to the high electrophilicity of the phosphorus center in RPE_2_ species (R = aryl/alkyl, E = O/S), where self-aggregation to cyclic or linear polymeric compounds was observed [53]. It is assumed that both mesomeric structures (shown in Scheme 4) can interact easily with carbonyls, alcohols, or heterocycles [54]. The reactivity of carbonyl derivatives in thionation by LR was ranked in the following order: amides > ketones > esters [55]. It was observed that the amide carbonyls could be selectively thionated with LR in the presence of esters and/or lactone groups [56]. The commonly accepted mechanism of carbonyls sulfurization consists of two steps (see Scheme 5) involving:

- A concerted cycloaddition of a reactive monomeric form of LR and the carbonyl-containing derivative to generate a four-membered thiaoxaphosphetane intermediate (**1**)
- A cycloreversion forming the corresponding thiocarbonyl analogue (**2**) and phenyl(thioxo)phosphine oxide (**3**).

The driving force of S→O substitution is the thermodynamic stability of the P=O bond formed in the product (**3**). In fact, the P–O bonds are stronger compared to their P–S counterparts; therefore, the formation of the thionated product seems privileged. On the whole, the mechanism of thiocarbonylation using the LR agent resembles the Wittig reaction with the formation of a phosphorus-based four-membered Wittig-like ring intermediate (**1**).

The worldwide popularity of LR in organic chemistry stems primarily from its marketed accessibility, the simplicity of its application, and the mild reaction conditions with high yields obtained in the shortened reaction time, especially while the synthesis is performed under solvent-free microwave irradiation (an economical and environmentally friendly methodology) [25]. Unluckily, the thio-substitution of carbonyl oxygen using LR under typical operational conditions has been criticized due to its propensity to form some stable heterocyclic intermediates and/or undesired (by-)products, particularly in the dry conditions of the aromatic hydrocarbon solvents at elevated temperatures [45,57]. Hence, the separation/purification of the reagent-derived (by-)products using simple extractive methods (e.g., filtration or distillation) seems to be an awkward task. In consequence, column chromatography on silica gel has to be employed. Due to the relatively high molecular weight of LR (MW = 404.47 g/mol), a fairly long chromatographic column should be applied, which makes the procedure unwieldy for anything but small-scale laboratory preparation [25,45]. The low-weighted molecular products can only be distilled directly from the reaction mixture. On the other hand, a range of alternative, high-boiling solvents (e.g., benzyl benzoate) was applied to the solid-phase parallel synthesis of thioamides [43]. The optimized, column-free thionation of amides in ethylene glycol solvent was proposed for scaling up the LR-based workup procedure [58]. Contrary to esters, the thionation of amides can be effectively accomplished in the environment of the anhydrous THF at room temperature [46]. Moreover, the microwave-accelerated thionating of amides improved noticeably not only the yields but significantly reduced the reaction time and simplified the workup and purification method [49]. Even the unique structural construction of the LR agent was modified by the introduction of fluorous ponytails in order to refine the isolation protocol of amide thionation, including the exclusion of the chromatographic columns [59]. The replacement of the methyl motif in the methoxy substituent (-OCH_3_) with a fluorous ponytail –(CH_2_)_4_(CF_2_)_6_F in the fluorous LR analogue (*f*-LR) increased the thioamide yields since a simple filtration (solid-phase extraction) was applied in the purification of the final reaction products. 

**Figure 3:**
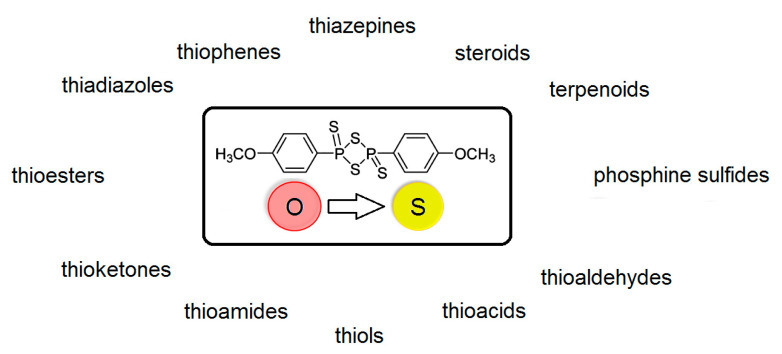
Selected organosulfur families prepared with Lawesson’s reagent.

It is still an open question whether LR is superior to P_4_S_10_ as a versatile thionating agent. LR has undoubtedly become an indispensable reagent for ‘sulfur’ chemistry with diverse applications for converting a range of oxo to thio functional groups, including some new classes of heterocycles, as shown in Figure 3. Having weighed up the pros and cons, the incorporation of sulfur atom(s) with the LR agent is currently favored by organic chemists, mainly due to better reaction yields obtained [25]. 

In order to develop ‘green’, eco-friendly sulfur transfer agents, the solid supports, as well as the ionic liquids, are being profoundly scrutinized. The detailed description of the other thionating agent is beyond the scope of this paper. 

## 2. Results and Discussion

### 2.1. Design and Dithioamide Synthesis

**Scheme 6:**
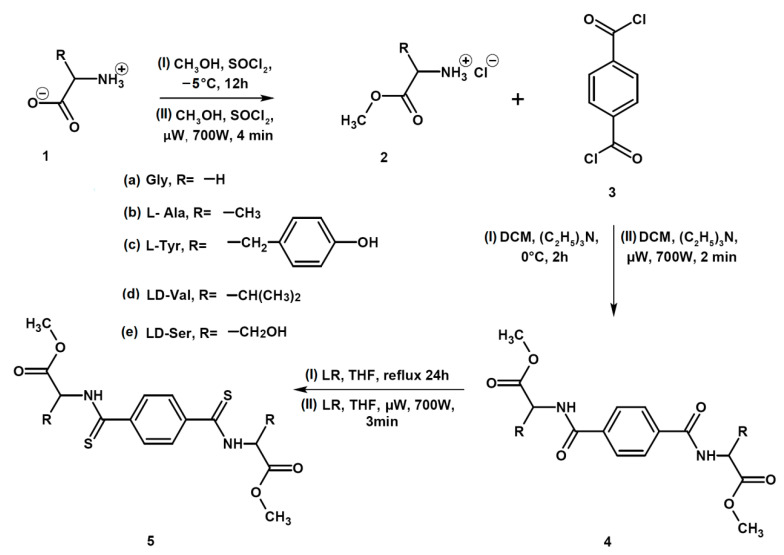
Synthesis of dithioamides (**5a**–**5e**) according to conventional (I) and microwave-supported (II) procedures.

Conceptually, the multistep synthesis of the symmetrical α-AA-based (R = Gly, Ala, Val, Tyr, and Ser) dithioamides of terephthalic acid was proposed using the conventional heating (method I) as well as the microwave-accelerated approach (method II). In fact, a comparative study on thionation yields of diamides with LR agent under conventional heating and microwave irradiation was conducted. The microwave-assisted methodology was successfully employed on the whole pathway from substrates (α-amino acids) to products (dithioamides of terephthalic acid) with a noticeably reduced reaction time/cost, simplified purification protocol, and increased reaction yields when compared to the older approach (method I). The small-scale treatment of the selected α-AA-based diamides of terephthalic acids using LR under microwave-aided conditions proceeded smoothly with satisfactory yields of the final products. The symmetrical dithioamides of terephthalic acid (**5a**–**5e**) were generated in a three-step synthesis, as illustrated in Scheme 6. In the first step, the primary amino and carboxylic moieties of the selected α-amino acid (**1**) were protected by a reaction with methyl alcohol and thionyl chloride to form an amino acid methyl ester hydrochloride (**2**). In the second step, involving amide bond formation, the reaction of α-AA-based methyl ester hydrochloride with the commercially available terephthalic acid dichloride (**3**) produced a series of symmetrical diamides (**4**). To protect the acid-sensitive amide functional group, trietyloamine was added to the methylene chloride (DCM) solvent. Finally, the sulfurization of the selected diamides was conducted with an LR agent in order to form the corresponding dithioamides (**5**). The final products were purified using column chromatography with a mixture of dichloromethane and ethyl acetate, respectively.

In an attempt to transform the oxo substituents (>C=O) of the symmetric diamides to the thio-functionalized (>C=S) counterparts, the unsubstituted (**a**), branched-chain (**b**,**d**), aromatic (**c**), and polar (**d**) α-AAs were selected as the substrates of the presented case study (see Scheme 6). In order to compare both methods quantitatively, exactly the same amounts of the substrates/solvents were used; however, the reaction time and the source of energy varied considerably. Obviously, the identical purification procedure was employed in both methods. Based on the reaction output (see Table 1), the microwave-accelerated synthetic route of the chosen α-AA-based dithioamides of terephthalic acid (**5a**-**5e**) yielded comparatively to the conventional reaction pathway. As a whole, at each stage of the (sub-)products (**2**), (**4**), and (**5**), the formation under microwave irradiation (method II) achieved yields slightly better compared to the older, traditional practice with the elongated reaction/heating time (method I). 

A significantly reduced reaction time (from days to minutes) and low energy requirements in the microwave-supported procedure compared to the traditional source of heating make the approach an attractive, resource/cost-optimized alternative to the lengthy methodology under conventional heating. 

### 2.2. Similarity-Oriented Property Evaluation

**Figure 4:**
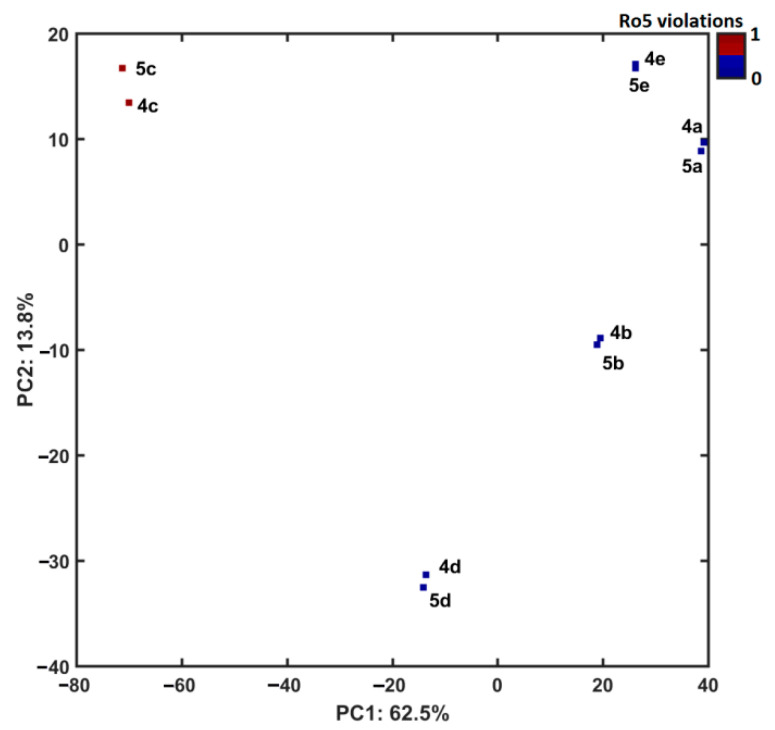
Projection of diamides (**4a**–**4e**) and dithioamides (**5a**–**5e**) of terephthalic acid on a plane defined by PC1 and PC2. Colors code the violations of Ro5 rule.

The clustering tendency of the descriptor-driven data can be investigated by tracing the (dis)similarities between objects (molecules **4a**–**4e**, **5a**–**5e**) in the multidimensional variable space [64]. Hence, the distance-related property assessment was performed using Principal Component Analysis (PCA) and Hierarchical Clustering Analysis (HCA) on the pool of 2757 descriptors generated by Dragon 6.0 software. The software-based data were organized into matrix X_10×2757_ with rows representing objects (compounds **4a**–**4e**, **5a**–**5e**) and columns representing numerical parameters (descriptors). The resulting matrix was centered and standardized because the calculated parameters differ considerably. The number of relevant principal components (PCs) was specified, taking into account the percentage of the modeled data variance. The first four PCs describe 92.1% of the total data variance, while the first two PCs account for 76.3%, respectively. The scoreplot with the projection of the corresponding diamides (**4a**–**4e**) and dithioamides (**5a**–**5e**) of terephthalic acid on the plane PC1 vs. PC2 color-coded according to Lipinski’s rule-of-5 (Ro5) violations is presented in Figure 4.

Not surprisingly, the diamides and the corresponding dithioamides of terephthalic acid are grouped together. Interestingly, Gly-, Ala-, and Ser-based molecules **4**,**5**(**a**,**b**,**e**) are clustered together along the first principal component (PC1 > 0), while Tyr-containing **4**,**5**(**c**) and Val-based **4**,**5**(**d**) compounds are separated from the remaining ones. Moreover, the structural dissimilarity between molecules **4**,**5**(**c**) and **4**,**5**(**d**) is also observed along the second principal component (PC2), where Tyr-containing compounds ‘break’ Lipinski’s Ro5 rule (MW > 500). 

**Figure 5:**
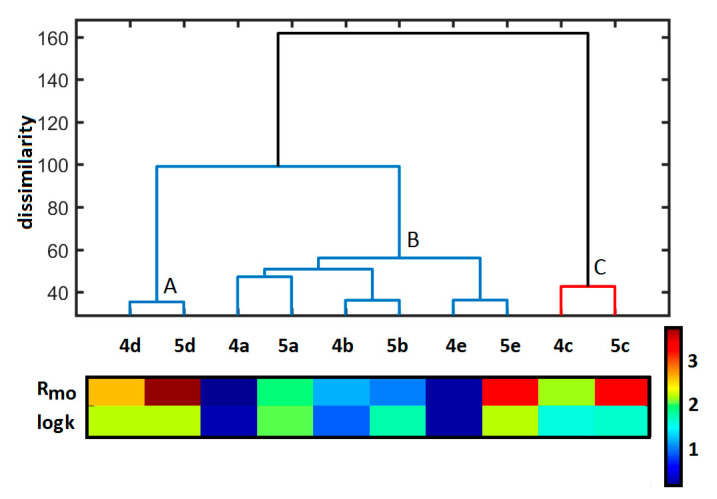
Dendrogram of diamides (**4a**–**4e**) and dithioamides (**5a**–**5e**) of terephthalic acid in descriptor-based space with a color-coded map of experimental lipophilic values (R_mo_ and log*k*).

Generally, the exploratory HCA approach produces the sub-optimal clustering pattern of objects that is largely dependent on the clusters’ linkage procedure employed [65]. In-depth interpretability of the multidimensional data is hampered in the original variable space; therefore, the results are represented as a dendrogram generated in the Euclidean-based distance with the Ward linkage algorithm—the OX axis illustrates the order of objects or parameters, while the OY axis presents the dissimilarity. The dendrogram presented in Figure 5 confirms our previous PCA findings (see Figure 4), where Tyr-containing molecules (cluster C) and Val-based ones (cluster A) differ from the rest of the objects (cluster B). Color-coded vectors of the experimental lipophilic data (TLC-based R_mo_ and HPLC-based log*k*) show that the molecules of cluster B are mainly characterized by lower values of R_mo_ and log*k* parameters, respectively. 

### 2.3. ClogP Approximation and Empirical Lipophilicity Investigation

**Figure 6:**
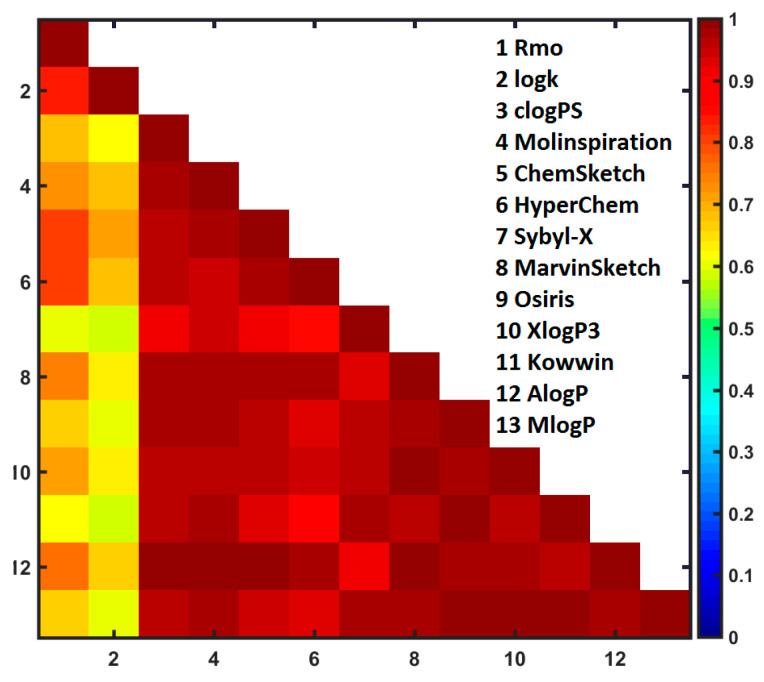
Matrix of correlation coefficients of linear relationships between experimental lipophilicity (R_mo_ and log*k*) and calculated lipophilicity (clogP) for diamides (**4a**–**4e**) and dithioamides (**5a**–**5e**) of terephthalic acid.

The approximation of the numerical clogP values for the investigated set of diamides (**4a**–**4e**) and dithioamides (**5a**–**5e**) of terephthalic acid was conducted using a range of in silico logP predictors, including AlogPS, Molinspirations, Osiris, HyperChem 7.0, Sybyl-X, MarvinSketch 15, ACD/ChemSketch 2015, Dragon6.0, Kowwin, and XlogP3. Moreover, the experimental investigation of the lipophilicity profile was performed using thin-layer chromatography (TLC) and high-performance liquid chromatography (HPLC), respectively. The theoretically estimated partition coefficients (clogP) were (inter-)correlated with each other and cross-compared with the empirically specified R_mo_ (TLC) and log*k* (HPLC) parameters, as shown in Figure 6.

A relatively good correlation (ranging from r = 0.60 to r = 0.81) between the generated clogP and the empirical R_mo_ value was revealed for all engaged clogP estimators, with r > 0.80 recorded for ChemSketch and HyperChem programs. A slightly worse correlation (r = 0.6÷0.7) was recorded between the calculated clogP and the experimental log*k* values. Despite some variations in clogP values, probably resulting from different computational algorithms (atom/fragment- or descriptor-based) implemented in the software and/or the training data used at the training step, a satisfactory inter-correlation between clogP predictors (r > 0.90) was noticed as well. 

## 3. Materials and Methods

### 3.1. General Methods

All reagents and solvents were purchased from Sigma-Aldrich, Chempur, Fluka, Avantor Performance Materials Poland S.A., and Thermo Fisher Scientific, respectively. Melting points were determined on an apparatus Stuart SMP10. Solvents were evaporated using a rotary evaporator IKA RV 10. Microwave-assisted syntheses were performed in RM800PC microwave laboratory reactor from Plazmatronika (Wroclaw, Poland) with monomode cavity, magnetic stirrer, and external IR temperature measurements. ^1^H-NMR spectra were recorded on a Bruker Avance 400 MHz spectrometer using DMSO-d_6_, CDCl_3_-d_6_, and ^13^C-NMR. Spectra were recorded on a Bruker Avance 101 MHz spectrometer using DMSO-d_6_ and CDCl_3_-d_6_. The chemical shifts (δ) are given in ppm, and multiplicities are given as s (singlet), d (doublet), t (triplet), q (quartet), and m (multiplet). The values of the coupling constants (J) are reported in hertz (Hz). IR spectrum Spektrometr FT-IR, SpectrumOne, Perkin Elmer was used. High-resolution mass spectra were measured using a high-performance liquid chromatograph Dionex UltiMate^®®^ 3000 (Thermo Scientific, Waltham, MA, USA) coupled with an LTQ Orbitrap XLTM Hybrid Ion Trap-Orbitrap Fourier Transform Mass Spectrometer (Thermo Scientific) equipped with a HESI II (heated electrospray ionization) source in the positive mode.

### 3.2. Synthesis of Dithioamides

#### 3.2.1. Method I: Conventional Procedure

A suspension (100 mmol) of the selected α-amino acids (AAs = Gly, Ala, Tyr, Val, and Ser) in 90 mL of anhydrous methanol was stirred with a magnetic stirrer under a reflux condenser and cooled in an ice bath to a temperature not exceeding −5 °C, and then thionyl chloride was added dropwise (250 mmol). Next, a homogeneous mixture was magnetically stirred overnight. After stirring, the solvent was evaporated in a vacuum, and a crude product was washed with diethyl ether, filtered through a Büchner funnel, and then dried over anhydrous calcium chloride. Then, the product was recrystallized using anhydrous methanol and filtered with active carbon. In the next step, the suspension of (20 mmol) amino acid methyl ester hydrochloride in 80 mL anhydrous dichloromethane was stirred with a magnetic stirrer and cooled in an ice bath below 0 °C, then 59.3 mmol of triethylamine was added dropwise, and the mixture was stirred for 20 min. Next, a suspension of (11.1 mmol) terephthalic acid dichloride in 30 mL dichloromethane was added dropwise, and the mixture was magnetically stirred for 2 h. Then, the solvent was evaporated in a vacuum, and the crude product was recrystallized using 200 mL of distilled water. Next, the mixture of (2.14 mmol) Lawesson’s reagent in 100 mL tetrahydrofuran at room temperature was added to the appropriate diamide. The reaction mixture was heated at the boiling point of the solvent for 24h. When the mixture was cooled to room temperature, the solvent was evaporated in a vacuum, and the product was purified on the column chromatography using dichloromethane: ethyl acetate (5:1) as the eluent.

#### 3.2.2. Method II: Microwave-Accelerated Procedure

A suspension (100 mmol) of the selected α-amino acids (AAs = Gly, Ala, Tyr, Val, and Ser) in 50 mL of anhydrous methanol was cooled in an ice bath to −5 °C, and then thionyl chloride was added dropwise (250 mmol). Next, a homogeneous mixture was exposed to microwave irradiation for 4 min. In the next step, the reaction mixture was transferred to an ultrasonic bath (30 °C, 15 min). Then, the solvent was evaporated in a vacuum, and a crude product was washed with diethyl ether, filtered through a Büchner funnel, and then dried over anhydrous calcium chloride. Next, the product was recrystallized using anhydrous methanol and filtered with active carbon. In the next step, the mixture of amino acid methyl ester hydrochloride (3.38 mmol) and (3.38 mmol) terephthalic acid dichloride in triethylamine and dichloromethane was exposed to microwave irradiation for 2 min. After that, the reaction mixture was transferred to an ultrasonic bath (30 °C, 10 min). Then, the solvent was evaporated in a vacuum, and a crude product was washed with distilled water and filtered through a Büchner funnel. Next, the mixture of the product and Lawesson’s reagent (1:1 mmol) in tetrahydrofuran and dichloromethane was exposed to microwave irradiation for 3 min. When the mixture cooled to room temperature, the reaction mixture was transferred to ultrasonic bath (30 °C, 15 min). Then, the solvent was evaporated in a vacuum, and the product was purified on the column chromatography using dichloromethane: ethyl acetate (5:1) as the eluent.

The chemical structures of the novel intermediates (symmetrical diamides **4c**–**4d**) and the final products (symmetrical dithioamides **5a**–**5e**) were characterized by ^1^H-NMR and ^13^C-NMR spectra (see Supplementary Materials). The melting points for new molecules were recorded as well.

1. *methyl 3-(4-hydroxyphenyl)-2-[(4-{[3-(4-hydroxyphenyl)-1-methoxy-1-oxopropan-2yl]carbamoyl}phenyl)formamido]propanoate* (**4c**) Mp 185 °C, ^1^H-NMR (400 MHz, DMSO) δ: 7.91 (s, 4H), 7.62- 7.40 (m, 8H), 6.83 (d, 2H), 4.31–3.96 (m, 2H), 3.83(d, 4H), 2.88 (s, 2H), 1.45 (s, 6H); ^13^C-NMR (101 MHz, DMSO) δ: 172.2, 166.6, 157.4, 133.4, 131.2, 128.25, 115.9, 53.8, 51.9, 40.6; 21.34;
2. *methyl 2-({4-[(1-methoxy-3-methyl-1-oxobutan-2-yl)carbamoyl]phenyl}formamido)-3-methylbutanoate* (**4d**) Mp 115 °C, ^1^H-NMR (400 MHz, DMSO) δ: 7.88 (s, 4H), 6.69 (d, 2H), 4.78 (ds, 2H), 3.79 (s, 6H), 2.29 (m, 2H), 1.01 (dd, 12H); ^13^C-NMR (101 MHz, DMSO) δ: 172.68, 166.52, 137.10, 127.57, 57.69, 52.52, 31.78, 19.13; 18.13;
3. *methyl 3-hydroxy-2-({4-[(3-hydroxy-1-methoxy-1-oxopropan-2-yl)carbamoyl]phenyl}formamido)propanoate* (**4e**) Mp 169 °C ^1^H-NMR (400 MHz, DMSO) δ: 8.08 (d, 2H), 7.73 (s, 4H), 4.14 (dd, 4H), 3.82 (s, 2H), 3.44 (s, 2H), 2.51 (s, 6H), ^13^C-NMR (101 MHz, DMSO) δ: 167.45, 132.06, 128.01, 67.89, 52.33, 45.76, 38.56;
4. *methyl 2-({4-[(2-methoxy-2-oxoethyl)carbamothioyl]phenyl}methanethioamido)acetate* (**5a**) Mp 86 °C, ^1^H-NMR (400 MHz, CDCl_3_)) δ: 8.40 (s, 2H), 7.81 (s, 4H), 4.60 (d, 4H), 3.84 (s, 6H); ^13^C-NMR (101 MHz, CDCl_3_) δ: 206.45 (C=S), 169.55 (C=O), 143.03, 126.99, 52.83, 47.58; IR (KBr) υ/cm^−1^: 3310, 1714, 1533, 1488, 1227, 1113; HR-MS: [M + H]+: calculated 341.425240 m/z, found 341.06986 m/z;
5. *methyl 2-({4-[(1-methoxy-1-oxopropan-2-yl)carbamothioyl]phenyl}methanethioamido)propanoate* (**5b**) Mp 174 °C, ^1^H-NMR (400 MHz, CDCl_3_) δ: 7.89 (s, 2H), 7.88 (s, 4H), 4.40 (t, 2H), 3.94 (d, 6H), 3.86 (s, 6H); ^13^C-NMR (101 MHz, CDCl_3_) δ: 197.29 (C=S), 171.54 (C=O), 135.27, 113.81, 67.88, 41.53, 37.55; IR (KBr) υ/cm^−1^: 3303, 1734, 1523, 1433, 1221, 1123; HR-MS: [M + H]+ C_14_H_16_N_2_O_4_S: calculated 369.47840 m/z, found 369.42786 m/z;
6. *methyl 3-(4-hydroxyphenyl)-2-[(4-{[3-(4-hydroxyphenyl)-1-methoxy-1-oxopropan-2-yl]carbamothioyl}phenyl)methanethioamido]propanoate* (**5c**) Mp 170 °C, ^1^H-NMR (400 MHz, DMSO) δ: 7.83 (d, 2H), 7.59 (s, 4H), 7.23–7.06 (m, 4H), 6.93–6.74 (m, 4H), 4.42 (s, 2H), 2.64–2.22 (m, 4H), 2.05 (2H), 1.55 (s, 6H); ^13^C-NMR (101 MHz, DMSO) δ: 190.74 (C=S), 165.68 (C=O), 136.32, 112.43, 62.45, 57.83, 54.67, 39.97, 37.12, 25.31, 14.90; IR (KBr) υ/cm^−1^: 3303, 1734, 1523, 1433, 1221, 1123; HR-MS: [M + H]+: calculated 553.66910 m/z, found 553.56812 m/z;
7. *methyl 2-({4-[(1-methoxy-3-methyl-1-oxobutan-2-yl)carbamothioyl]phenyl}methanethioamido)-3-methylbutanoate* (**5d**) Mp 160 °C, ^1^H-NMR (400 MHz, D_2_O) δ: 8.10 (d, 2H), 7.91 (s, 4H), 4.82 (dd, 2H), 3.78 (s, 6H), 2.30 (m,2H), 1.02 (t, 12H); ^13^C-NMR (101 MHz, D_2_O) δ: 190.71 (C=S), 167.58 (C=O), 137.12, 125.60, 57.78, 52.60, 32.05, 19.13, 18.25; IR (KBr) υ/cm^−1^: 3275, 1717, 1532, 1412, 1215, 1157; HR-MS: [M + H]+: calculated 425.58470 m/z, found 425.58482 m/z;
8. *methyl 3-hydroxy-2-({4-[(3-hydroxy-1-methoxy-1-oxopropan-2-yl)carbamothioyl]phenyl}methanethioamido)propanoate* (**5e**) Mp 179 °C, ^1^H-NMR (400 MHz, DMSO) δ: 8.49 (d, 2H), 7.88 (s, 4H), 4.02 (s, 2H), 3.91–3.06 (m, 2H), 2.51 (s, 6H), 2.12–1.80 (m, 2H), 1.72– 1.15 (m, 2H); ^13^C-NMR (101 MHz, DMSO) δ: 190.74 (C=S), 157.83 (C=O), 54.67, 39.97, 25.31, 14.90, 14.5; IR (KBr) υ/cm^−1^: 3402, 3286, 1584, 1432, 1316, 1128; HR-MS: [M + H]+: calculated 402.48570 m/z, found 402.39668 m/z.

### 3.3. X-ray Crystallography

**Figure 7:**
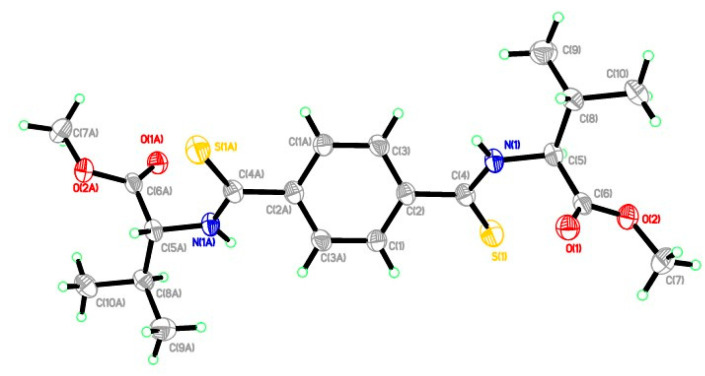
Molecular structure of compound **5d**.

*X*-ray analysis not only confirmed the structure of the final products determined by spectroscopic methods but also showed their spatial structure in the solid phase. Via crystallization from chloroform:ethanol (1:1 (*v*/*v*)), obtaining a single crystal of the derivative **5d** was possible. The X-ray structure of the derivative **5d** is shown in Figure 7, while its crystal data and structure refinement are given in Table 2. 

The selected bond lengths and angles are summarized in Table 3. 

### 3.4. Experimental Lipophilicity Specification 

#### 3.4.1. TLC-Based Lipophilicity Determination

The lipophilic studies were performed on the chromatographic plates for RP-TLC analysis purchased from Merck (Darmstadt, Germany): TLC Silica gel 60 RP-18 F_254S_. The lipophilicity determination of the investigated compounds was carried out on chromatographic plates 10 × 10 cm developed using mobile phases (50 mL) that were prepared by mixing the respective amounts of the organic modifier methanol and water. In the case of the organic modifier, concentrations (volume fraction, *v*/*v*) varied in a range from 0.60 to 1.00 in constant steps of 0.10. Using glass capillaries, 5 drops of compounds were applied on the same chromatographic plates. Chromatography was performed in a classical developing chamber, which was previously saturated with mobile phase vapors for 30 min. The migration distance was 7.0 cm, which takes about 20 min for the complete development of the chromatographic plates. Next, the plates were dried at room temperature (23 ± 1 °C) and visualized in UV light (λ = 254 nm). All analyses were repeated in triplicate in order to calculate the averaged values of R_F_ (retardation factor) that were subsequently converted into R_M_ values.

#### 3.4.2. HPLC-Based Lipophilicity Determination

For chromatographic analysis, stock solutions (50 µg/mL) of each test sample were prepared in acetonitrile (LiChrosolv^®^, Merck, Darmstadt, Germany). Chromatographic measurements were performed using the ACQUITY UPLC I-Class system (Waters) with a Xevo G2-XS QTof, ESI + detector, and an auxiliary PDA detector (λ = 254 nm). ACQUITY UPLC BEH C18 columns, 130 Å, 1.7 µm, 2.1 mm × 100 mm (Waters Corp.) were used for separation, operating at 35 °C, flow of 0.35 mL/min, and sample injection of 1 µL. The retention behavior of the analytes as a function of the mobile phase composition range (acetonitrile, LiChrosolv^®^, Merck + 0.01% HCOOH—water, LiChrosolv^®^, Supelco + 0.01% HCOOH) was tested. The concentration of acetonitrile, expressed as *v/v* by volume, varied from 0.25 to 0.50 in constant steps of 0.05. HCOONa solution (20 µg/mL) was used as a reference substance. The components of the analyte were identified on the basis of the monoisotopic masses of molecular ions [M + H]^+^ determined with an error of <5 ppm. Capacity factors *k* were calculated according to the formula *k* = (t_R_ − t_D_)/t_D_, where t_R_ is the retention time of the solute, and t_D_ is the dead time obtained using an unretained analyte. Each experiment was repeated three times. Log*k*, which was calculated from the capacity factor *k*, was used as the lipophilicity index, converted to the logP scale. 

#### 3.4.3. X-ray Crystallography

The X-ray data for colourless crystal thioamides was obtained at 120 K using a Gemini A Ultra (firmy Oxford Diffraction) with a CCD detector, using MoK_α_ radiation (λ = 0.71073 Å). The data collection and reduction were realized using the SCALE3 ABSPACK software package (CrysAlis RED, Oxford Diffraction Ltd., Version 1.171.37.35g). All hydrogen atoms were found in the difference Fourier maps and refined using a riding model with C–H = 0.95 Å for (CH)_aromatic_ and 0.98 Å for (CH_3_), and with U_iso_(H) = 1.2 U_eq_(CH) and 1.5 U_eq_(CH_3_). The N–H and O–H hydrogen atoms were refined using a similar manner with the AFIX 43 and AFIX 83 instruction, respectively. The graphics were drawn, and additional structural calculations were performed using Olex2 SHELXS and SHELXL.

Crystallographic data have been deposited with the Cambridge Crystallographic Data Centre under CCDC deposition number: 1060923. Copies of this information may be obtained free of charge from the Director, CCDC, 12 Union Road, Cambridge CB2 1EY, UK (fax: +44-1223-336033; e-mail: deposit@ccdc.cam.ac.uk).

## 4. Conclusions

In summary, the multistep synthesis of the novel α-AA-based dithioamides of terephthalic acid was proposed using conventional heating (method I) as well as the microwave-assisted approach (method II). A comparative study on thionation yields of the symmetrical diamides with Lawesson’s reagent (LR) under conventional heating and microwave irradiation was conducted. The microwave-accelerated methodology was successfully employed on the whole pathway from the chosen substrates (Gly, Ala, Val, Tyr, and Ser) to products (α-AA-based dithioamides of terephthalic acid) with a significantly reduced reaction time, energy requirements, and slightly increased reaction yields when compared to the older approach (method I). It should be emphasized that the small-scale treatment of the selected α-AA-based diamides of terephthalic acids using LR under microwave-supported conditions proceeded smoothly with satisfactory yields of the final products. Thus, the high-yield synthetic pathway to transfer the symmetric diamides of terephthalic acids to thio-substituted analogues under microwave irradiation was presented (method II). The symmetrical dithioamides of terephthalic acid were generated in a three-step synthesis. The chemical structures of the new intermediates and the final products (**5a**–**5e**) were characterized by ^1^H-NMR and ^13^C-NMR spectra. Moreover, the intermolecular similarity of novel terephthalic acid derivatives was estimated in the multidimensional space (mDS) of the structure/property-related in silico descriptors using principal component analysis (PCA) and hierarchical clustering analysis (HCA), respectively. Hence, the distance-oriented property distribution for the congeneric series of compounds was correlated with the experimental TLC and HPLC lipophilic data. Interestingly, Gly-, Ala-, and Ser-based molecules are clustered together along the first principal component (PC1 > 0), while Tyr-containing and Val-based compounds are separated from the remaining ones. Moreover, the structural dissimilarity between the above molecules is also observed along the second principal component (PC2), where Tyr-containing compounds ‘break’ the Lipinski’s Ro5 rule (MW > 500). The HCA findings confirmed the PCA results, where Tyr-containing molecule (cluster C) and Val-based compounds (cluster A) differ from the rest of the objects (cluster B). Color-coded vectors of the experimental lipophilic data (TLC-based R_mo_ and HPLC-based log*k*) show that molecules of cluster B are mainly characterized by lower values of R_mo_ and log*k* parameters. In fact, a relatively good correlation between the generated clogP and the empirical R_mo_ value was revealed for all engaged clogP estimators. A slightly worse correlation was recorded between the calculated clogP and the experimental log*k* values. Finally, the crystal structure of compound (**5d**) is provided as well.

As a matter of fact, the introduction of soft-donor sulfur into amide carbonyl oxygen of α-AA-based diamides can increase the complexation potency and selective recognition of biologically relevant transition metal ions. In consequence, dithioamides of terephthalic acid can potentially create ADMET-friendly conglomerates with drug molecules and serve as attractive drug carriers for poorly absorbed curative agents. 

## Figures and Tables

**Table 1:** The yields of (sub-)products in the synthesis of dithioamides of terephthalic acid under conventional heating (method I) and microwave-aided conditions (method II), expressed as the mean value (*n* = 2 experiments) [60,61,62,63].

Yield (%)									
		Method I				Method II			
**L.p.**	**R**	**2 ^a^**	**4 ^a^**	**5 ^a^**	**t ^a^ (h)**	**2 ^b^**	**4 ^b^**	**5 ^b^**	**t ^b^ [min]**
**1a**	-H	70%	53%	75%	38	85%	56%	78%	9
**1b**	-CH_3_	83%	64%	60%	38	87%	65%	71%	9
**1c**	-CH_2_C_4_H_6_OH	66%	58%	62%	38	70%	58%	65%	9
**1d**	-CH(CH_3_)_2_	90%	84%	42%	38	92%	41%	45%	9
**1e**	-CH_2_OH	73%	59%	48%	38	82%	63%	43%	9

^a^ conventional heating; ^b^ microwave irradiation; t—time of synthesis.

**Table 2:** Crystal data and structure refinement for molecule **5d**.

Formula	C_20_H_28_N_2_O_4_S_2_
Formula weight	424.56
Temperature	295(2) K
Crystal system	Triclinic
Space group	*P-1*
Unit cell dimensions	*a* = 7.7129(8) Å *α* = 69.793(6)°
	*b* = 8.9094(6) Å *β* = 67.042(8)°
	*c* = 9.5907(6) Å *γ* = 85.614(7)°
Volume	568.20(8) Å^3^
*Z*	1
Density (calculated)	1.241 mg/m^3^
Absorption coefficient	0.261 mm^−1^
F(000)	226
Crystal size	0.320 × 0.160 × 0.080 mm
Theta range for data collection	3.49 to 25.24°
Index ranges	−9 ≤ h ≤ 10, −11 ≤ k ≤ 11, −12 ≤ l ≤ 13
Reflections collected	5465
Independent reflections	4381 [R(int) = 0.0288]
Data/restraints/parameters	2660/0/130
Goodness-of-fit on *F*^2^	1.048
Final *R* indices [I > 2σ(I)]	*R*1 = 0.0496, *wR2* = 0.1073
*R* indices (all data)	*R*1 = 0.0841, *wR*2 = 0.1289
Largest diff. peak and hole	0.265 and −0.220 e.Å^−3^

**Table 3:** Lengths of bonds and angles (Å) of molecule **5d**.

S1–C4	1.654(2)	C8–C10	1.524(3)
O1–C6	1.202(2)	O2–C6	1.327(3)
O2–C7	1.445(3)	N1–C4	1.334(3)
N1–C5	1.450(3)	C1–C2	1.387(3)
C1–C3_a	1.379(3)	C2–C4	1.488(3)
C2–C3	1.390(3)	C5–C6	1.516(3)
C5–C8	1.539(3)	C8–C9	1.519(4)
C6–O2–C7	115.87(17)	C4–N1–C5	123.96(18)
C5–N1–H1	118.00	C4–N1–H1	118.0
C2–C1–C3_a	120.9(2)	C1–C2–C3	118.51(18)
C3–C2–C4	121.17(18)	C1–C2–C4	120.3(2)
C1_a–C3–C2	120.58(18)	S1–C4–C2	122.06(16)
S1–C4–N1	123.40(16)	N1–C4–C2	114.5(2)
N1–C5–C8	109.24(18)	N1–C5–C6	108.93(14)
C6–C5–C8	110.80(16)	O1–C6–C5	124.2(2)
O2–C6–C5	111.55(15)	O1–C6–O2	124.1(2)
C9–C8–C10	111.29(18)	C5–C8–C9	110.02(18)
C5–C8–C10	111.3(2)		

## Data Availability

Data is contained within the article and supplementary material.

## Supplementary Materials

The following supporting information can be downloaded at: https://www.mdpi.com/article/10.3390/ph16070984/s1, Figures S1–S8: H^1^ NMR and C^13^ NMR data.

## Abbreviations

Gly	Glycine.
Ala	Alanine.
Val	Valine.
Tyr	Tyrosine.
Ser	Serine.
DCM	Dichloromethane.
THF	Tetrahydrofuran.
