# Supplementary material for: Towards Symmetric Thioamides: Microwave-Aided Synthesis of Terephthalic Acid Derivatives
Source: Pharmaceuticals (Basel). 2023 Jul 9;16(7):984. doi: 10.3390/ph16070984 (PMC10385826; doi:10.3390/ph16070984)

Supplementary Materials

Toward symmetric thioamides: Microwave-aided synthesis of terephthalic acid derivatives

Andrzej Bak [1,*], Violetta Kozik [1,*], Aleksandra Swietlicka [1], Wojciech Baran [2], Adam Smolinski [3] and Andrzej Zieba [4]

[1] Institute of Chemistry, University of Silesia, Szkolna 9, 40-006 Katowice, Poland; aswietlicka@us.edu.pl
[2] Department of General and Analytical Chemistry, Faculty of Pharmaceutical Sciences in Sosnowiec, Medical University of Silesia in Katowice, Jagiellońska 4, 41-200 Sosnowiec, Poland; wbaran@sum.edu.pl
[3] Central Mining Institute, Plac Gwarków 1, 40-166 Katowice, Poland; smolin@gig.katowice.pl
[4] Department of Organic Chemistry, Faculty of Pharmaceutical Sciences in Sosnowiec, Medical University of Silesia in Katowice, Jagiellońska 4, 41-200 Sosnowiec, Poland; zieba@sum.edu.pl
* Correspondence: andrzej.bak@us.edu.pl (A.B.); violetta.kozik@us.edu.pl (V.K.); Tel.: +48-32-359-11-97 (A.B.)

Figure S1. H^1 NMR and C^{13} NMR spectra of compound **4c**.

^{1}H NMR

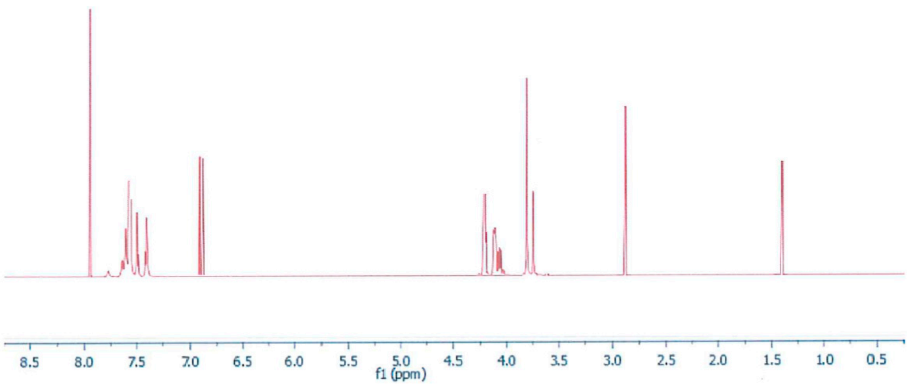

^{13}C NRM

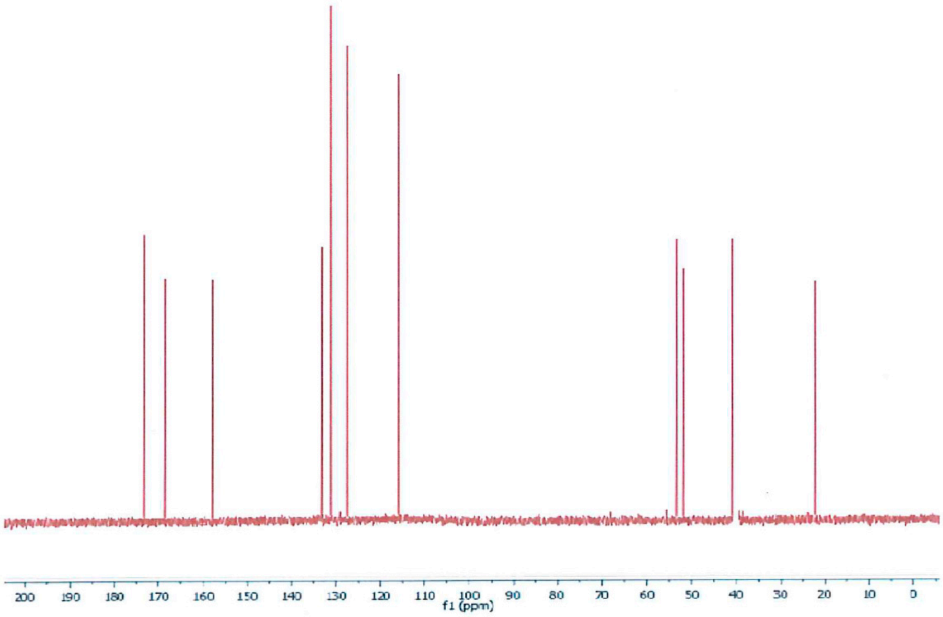

Figure S2. H^1 NMR and C^{13} NMR spectra of compound **4d**.

^{1}H NMR

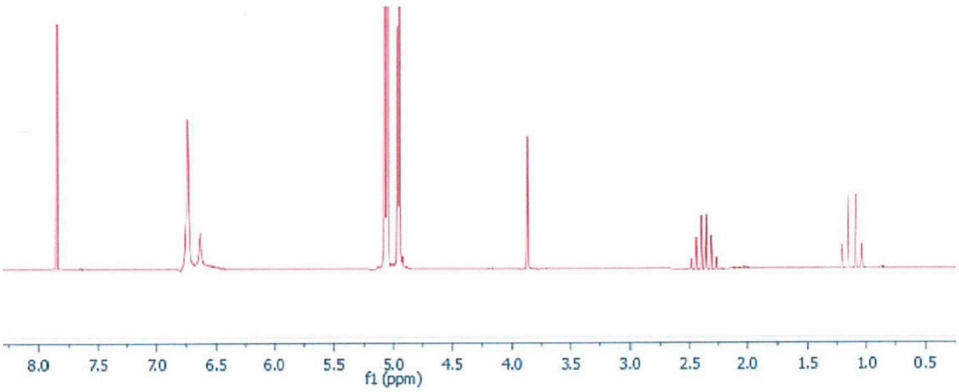

^{13}C NMR

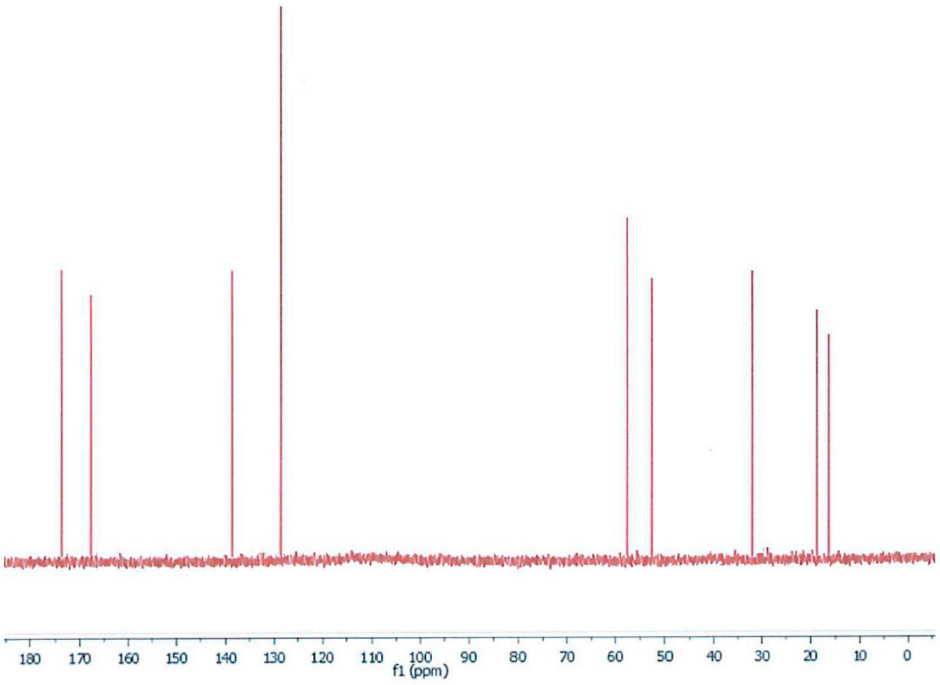

Figure S3. H^1 NMR and C^{13} NMR spectra of compound **4e**.

^{1}H NMR

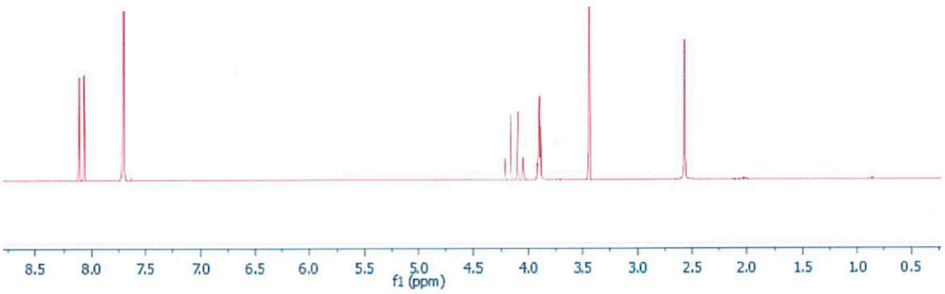

^{13}C NMR

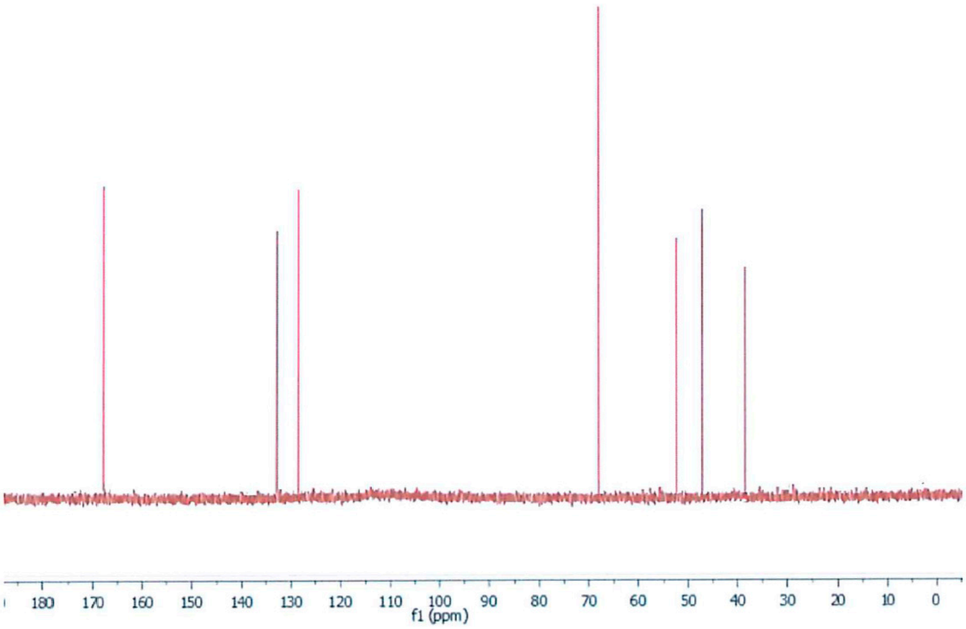

Figure S4. H^1 NMR and C^{13} NMR spectra of compound **5a**.

^1H NMR

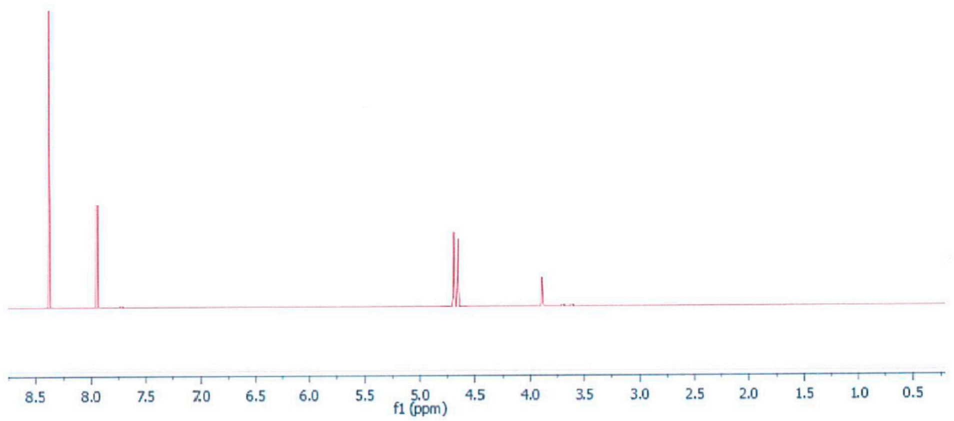

^{13}C NMR

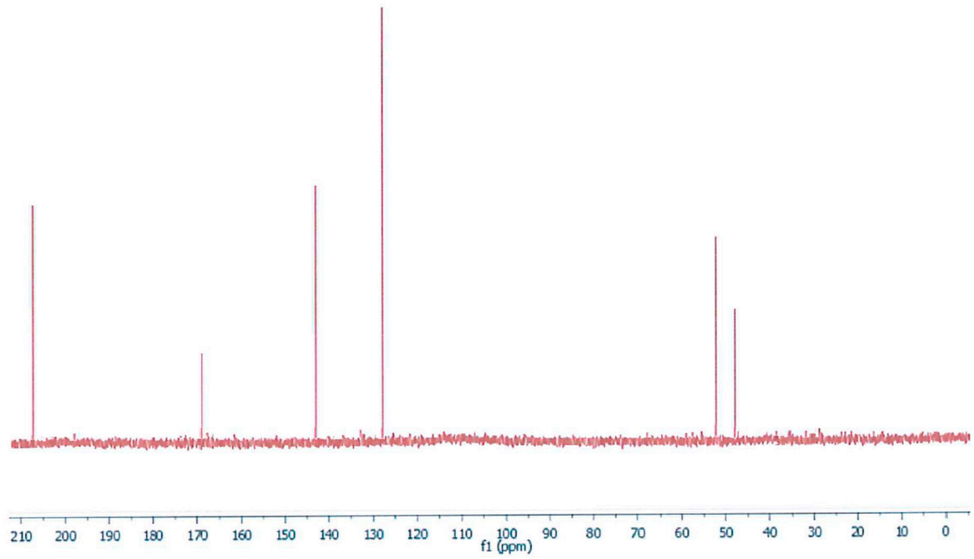

Figure S5. H^1 NMR and C^{13} NMR spectra of compound **5b**.

^{1}H NMR

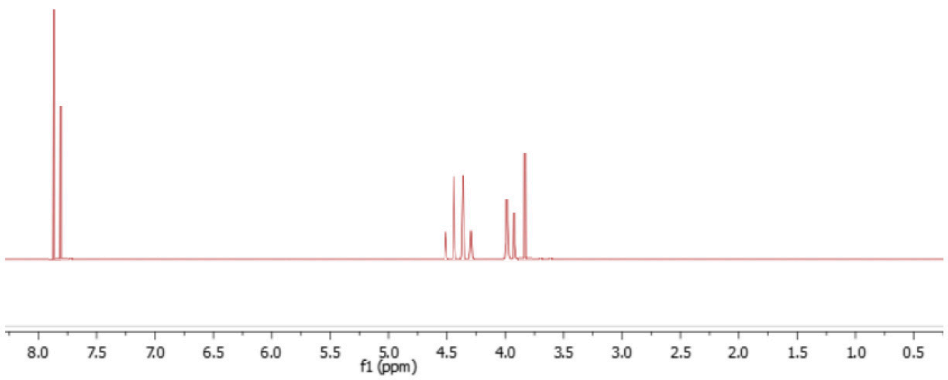

^{13}C NMR

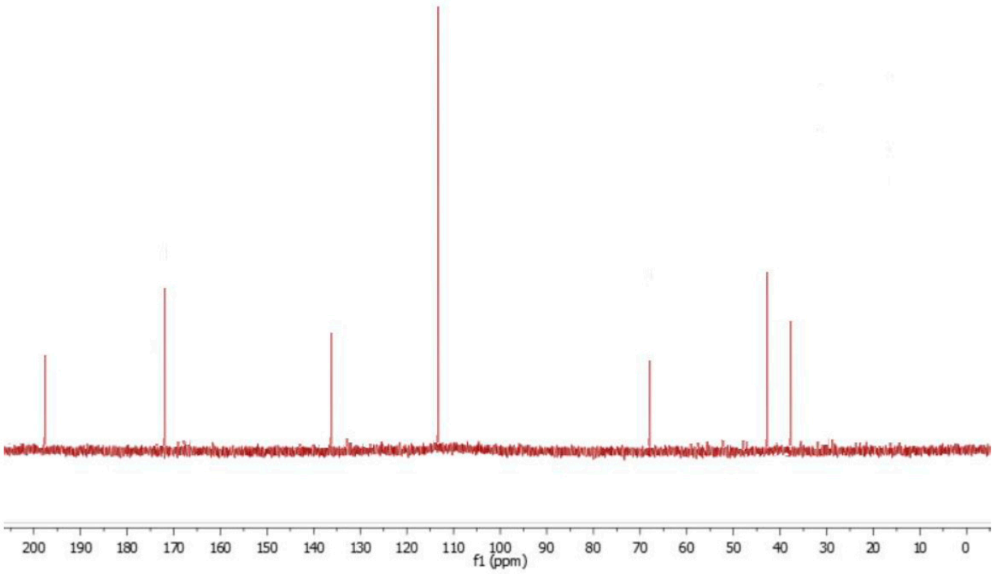

Figure S6. H^1 NMR and C^{13} NMR spectra of compound **5c**.

^{1}H NMR

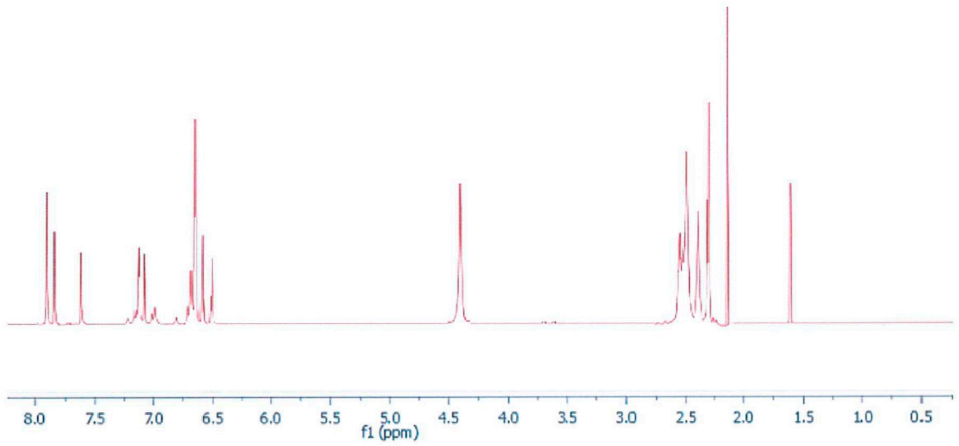

^{13}C NMR

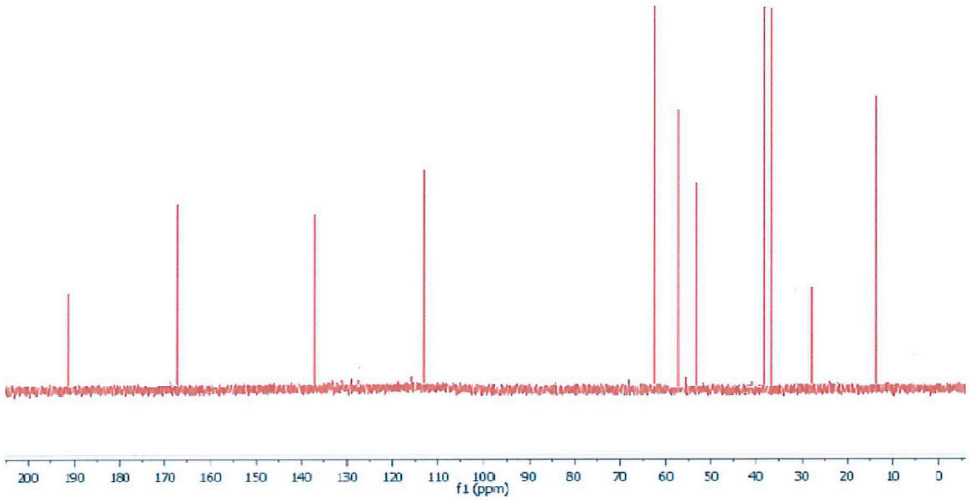

Figure S7. H^1 NMR and C^{13} NMR spectra of compound **5d**.

^{1}H NMR

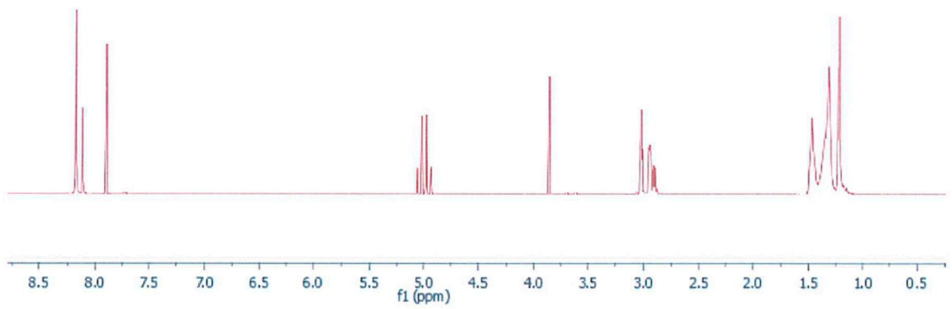

^{13}C NMR

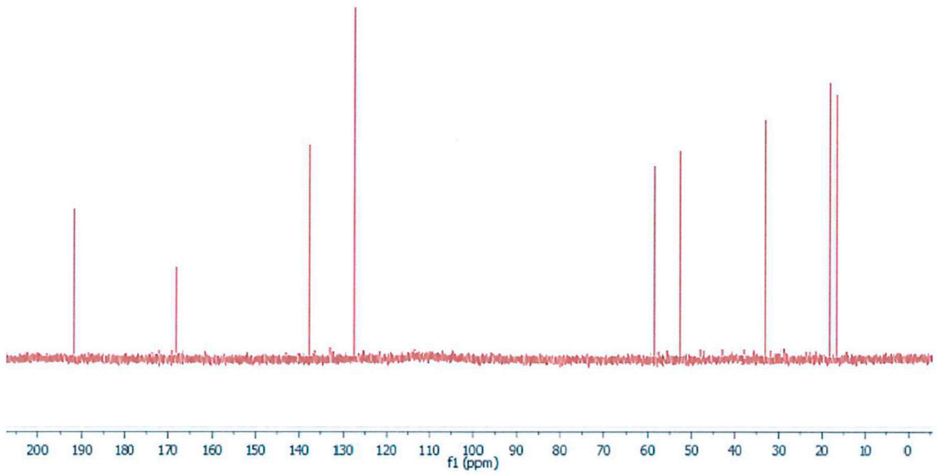

Figure S8. H^1 NMR and C^{13} NMR spectra of compound **5e**.

^{1}H NMR

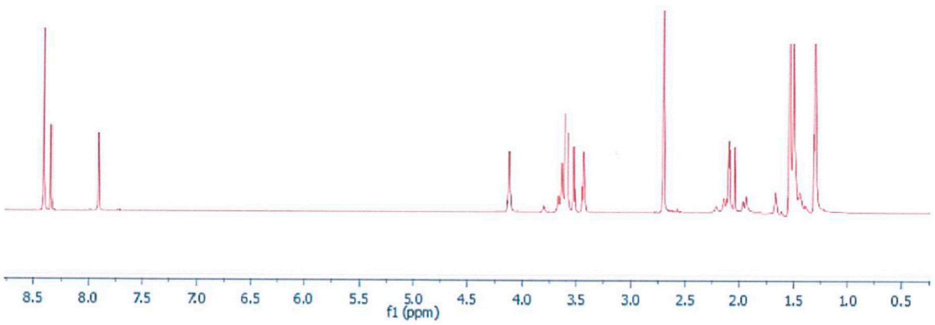

^{13}C NMR

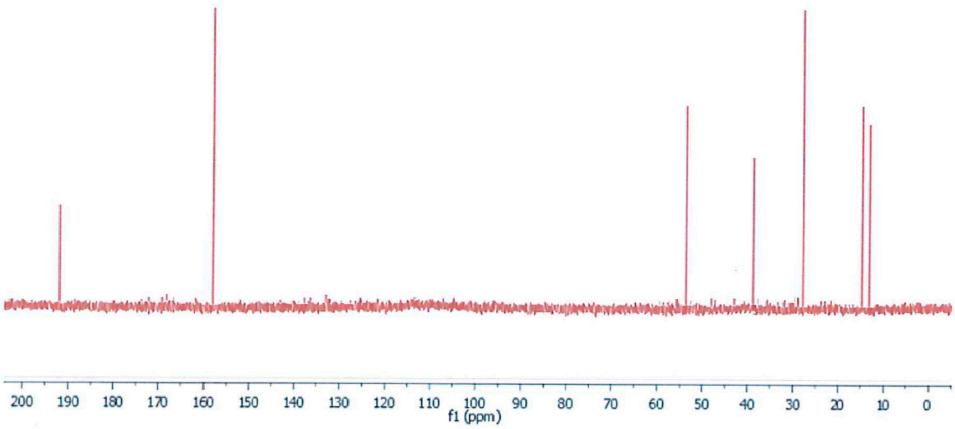

Supplement: Supplementary file 1 [file pharmaceuticals-16-00984-s001.zip › pharmaceuticals-2495108-supplementary.pdf]
